# Secondary Prevention of Hip Fragility Fractures During the COVID-19 Pandemic: Service Evaluation of “MRS BAD BONES”

**DOI:** 10.2196/25607

**Published:** 2020-12-22

**Authors:** Alastair Stephens, Hannah Rudd, Emilia Stephens, Jayne Ward

**Affiliations:** 1 Trauma and Orthopaedic Department University Hospital Coventry and Warwickshire Coventry United Kingdom

**Keywords:** osteoporosis, fragility fracture, guideline, mnemonic, acronym, COVID-19, bone, morbidity, mortality, fracture, elderly, older adults, geriatrics, audit, prevention

## Abstract

**Background:**

Management of osteoporosis is an important consideration for patients with femoral neck fractures due to the morbidity and mortality it poses. The input of orthogeriatric teams is invaluable in coordinating secondary fragility fracture prevention. The COVID-19 pandemic resulted in the rapid restructuring of health care teams and led to the redeployment of orthogeriatricians.

**Objective:**

This study aimed to determine the impact COVID-19 had on the secondary prevention of fragility fractures among patients with femoral neck fractures, and to optimize management in this population.

**Methods:**

A retrospective audit was conducted of patients with femoral neck fractures before and after the lockdown in response to the COVID-19 pandemic in the United Kingdom. A reaudit was conducted following the development of our new mnemonic, “MRS BAD BONES,” which addressed key factors in the assessment and management of osteoporosis: *m*edication review, *r*heumatology/renal advice, smoking cessation; *b*lood tests, *a*lcohol limits, *D*EXA (dual energy X-ray absorptiometry) scan; *b*one-sparing medications, *o*rthogeriatric review, *n*utrition, *e*xercise, supplements. The Fisher exact test was used for comparison analyses between each phase.

**Results:**

Data for 50 patients were available in each phase. The orthogeriatric team reviewed 88% (n=44) of patients prelockdown, which fell to 0% due to redeployment, before recovering to 38% (n=19) in the postintervention period. The lockdown brought a significant drop in the prescription of vitamin D/calcium supplements from 81.6% (n=40) to 58.0% (n=29) (*P*=.02); of bone-sparing medications from 60.7% (n=17) to 18.2% (n=4) (*P*=.004), and DEXA scan requests from 40.1% (n=9) to 3.6% (n=1) (*P*=.003). Following the implementation of our mnemonic, there was a significant increase in the prescription of vitamin D/calcium supplements to 85.7% (n=42) (*P*=.003), bone-sparing medications to 72.4% (n=21) (*P*<.001), and DEXA scan requests to 60% (n=12) (*P*<.001).

**Conclusions:**

The redeployment of the orthogeriatric team, due to the COVID-19 pandemic, impacted the secondary prevention of fragility fractures in the study population. The “MRS BAD BONES” mnemonic significantly improved management and could be used in a wider setting.

## Introduction

Osteoporosis, characterized by the progressive degradation of the microarchitecture of bone tissue and resultant loss in bone density, is a leading cause of femoral neck fractures in the elderly [1]. In the United Kingdom, there are approximately 536,000 new fragility fractures each year, of which 79,000 are femoral neck fractures [2]. This is a significant cause of increased morbidity and mortality among the elderly, with an average cost of treatment to the National Health Service (NHS) estimated at £12,000 (US $16,311) per patient [3,4]. Furthermore, patients who suffer an osteoporotic fracture are at a greater risk of sustaining a second osteoporotic fracture [3].

The management of femoral neck fractures has evolved over time. Best practice tariffs have been established to optimize the care of these patients, which should include postoperative rehabilitation and assessment for secondary fracture risk under the guidance of geriatrician-directed multidisciplinary teams [5]. Orthogeriatric comanagement has significantly reduced 30-day mortality in patients with hip fractures from 13.4% to 10.3% [6], with positive influence on functional outcomes and future fracture risk [7]. The National Institute for Health and Care Excellence (NICE) guidelines specifically support the management of bone health during admission for femoral neck fractures and recommend initiating bisphosphonate therapy (or an alternative) in addition to calcium and vitamin D supplementation to all patients with fragility fractures [8].

This year has brought an unprecedented challenge to health care services worldwide, with the declaration of the global pandemic of SARS-CoV-2 on March 11, 2020 [9]. The pandemic resulted in the rapid restructuring of health care teams to respond to the large influx of medically unwell patients across hospital sites. Within our Trauma and Orthopaedic (T&O) department, this resulted in the redeployment of the orthogeriatric team, who had previously been responsible for coordinating the management of secondary prevention of fragility fractures in patients with femoral neck fractures.

This project was part of a quality improvement initiative. The aim of this study was to determine the impact COVID-19 had on the secondary prevention of fragility fractures in patients with femoral neck fractures, and to optimize management in this population.

## Methods

All requirements to carry out the study were sent to the research and development department to assess risks to patient identification. The department approved the study (ID number: 698), and confirmed that as the project was a local audit without use of patient identifiable information, there was no need for further ethical approval. This study was based on patients with femoral neck fractures admitted to the major trauma center at University Hospital Coventry and Warwickshire (UHCW) between February 2020 and June 2020. Patients were recruited consecutively by admission date, and the same inclusion and exclusion criteria were applied. The inclusion criteria involved all patients with a low-energy femoral neck fracture aged ≥60 years on admission. The exclusion criteria involved patients with periprosthetic fractures, patients who were managed nonoperatively, patients who were taking bone-sparing treatment preadmission, patients for whom it was documented as inappropriate to commence bone-sparing treatment due to a palliative approach, and patients who died while in hospital.

Data were collected in 2 phases from 50 consecutively admitted patients; the first phase recruited patients admitted immediately prior to the national UK lockdown on March 23, 2020, to determine the department’s baseline compliance. The second phase refers to those admitted immediately after the lockdown.

**Figure 1:**
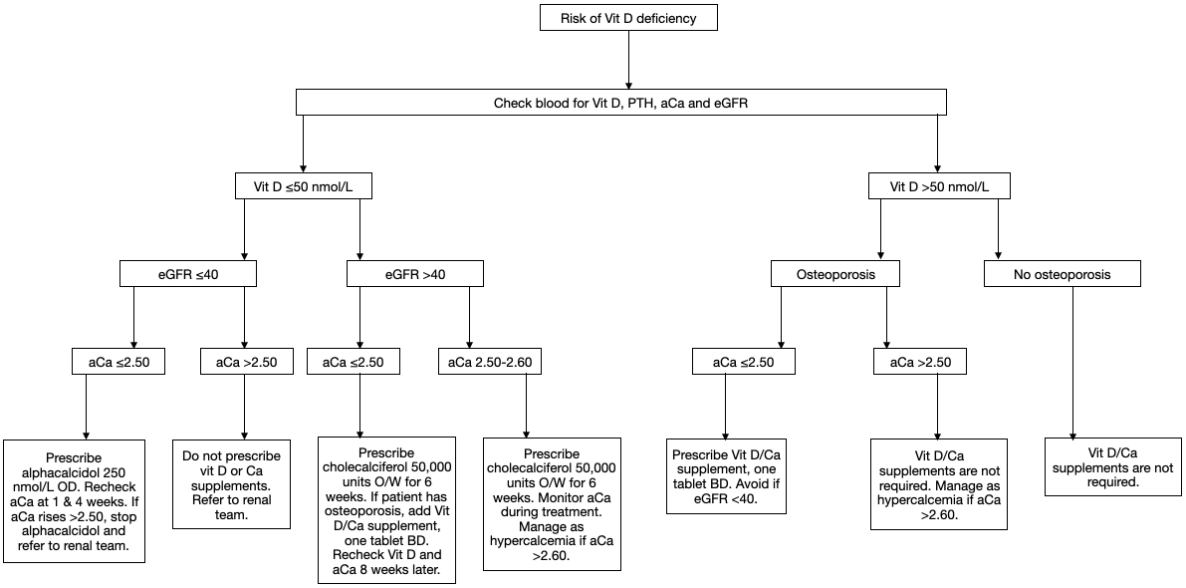
Assessment and management of patients with femoral neck fractures at risk of vitamin D deficiency, as described by the University Hospital of Coventry and Warwickshire guidelines. Vit D: vitamin D; eGFR: estimated glomerular filtration rate; PTH: parathyroid hormone; aCa: adjusted calcium, OD: once daily; O/W: once weekly; BD: twice daily.

**Figure 2:**
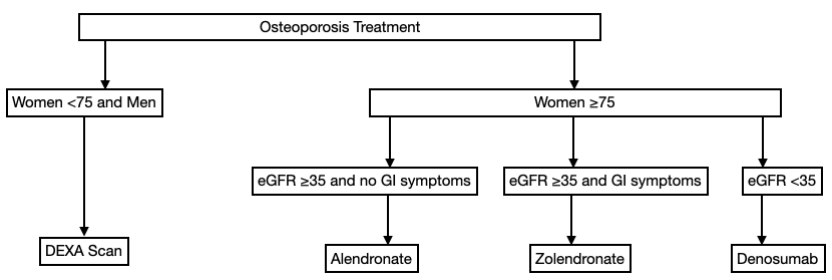
Osteoporosis assessment and treatment for patients with femoral neck fractures as per the University Hospital of Coventry and Warwickshire guidelines. eGFR: estimated glomerular filtration rate; DEXA: dual energy X-ray absorptiometry; GI: gastrointestinal.

All patients were assessed as to whether the secondary prevention of fragility fractures had been carried out upon discharge as described by UHCW trust guidelines. Figure 1 describes the local guidelines used for managing vitamin D and calcium deficiency in illustrative form. This involved measuring serum vitamin D, parathyroid hormone (PTH), adjusted calcium, and estimated glomerular filtration rate (eGFR), followed by prescribing appropriate supplements [10]. The frequencies and percentages of patients who had these parameters measured were recorded. Figure 2 demonstrates the osteoporosis treatment local guidelines in the femoral neck fracture population. Women under 75 years of age and all men met the criteria for dual energy X-ray absorptiometry (DEXA). The DEXA scan service continued as normal during the lockdown period. Women aged ≥75 years met the criteria for bone-sparing medications, taking into consideration gastrointestinal side effects and renal function.

Available case notes, primarily orthogeriatric review, drug charts, discharge summaries, DEXA scan request forms, and laboratory results, were reviewed, with local laboratory parameters used. The lowest measurable value in our laboratory for vitamin D serum levels is <10 nmol/L, so for the purposes of analysis, this was substituted for a value of 10 nmol/L. eGFR was calculated using the Modification of Diet in Renal Disease equation (ml/min/1.73 m^2^). Adjusted calcium in mg/dL was calculated using (0.8 × [normal albumin – patient’s albumin]) + serum calcium level. A normal albumin level was defaulted to 40 g/L.

For these first 2 phases, data were collected retrospectively using information available from electronic patient records. Consequently, we were only able to assess orthogeriatric reviews, blood test results, DEXA scan requests, bone-sparing medications, and supplements prescribed.

The results of the review were presented at the monthly T&O quality improvement projects meeting. A new mnemonic, “MRS BAD BONES,” was developed as a tool to improve junior doctor, advanced nurse practitioner, and medical student awareness of secondary fragility fracture prevention. The mnemonic represents the following:

- Medication review
- Rheumatology/renal advice
- Smoking cessation
- Blood tests: calcium, eGFR, PTH, and vitamin D
- Alcohol limits
- DEXA scan
- Bone-sparing medications: bisphosphonates
- Orthogeriatric review
- Nutrition
- Exercise
- Supplements: calcium and vitamin D

A third and final phase of data was then collected prospectively following the dissemination of the acronym at the quality improvement projects meeting. Due to the limitations of data available from phases 1 and 2, we decided to only measure the same 5 parameters in phase 3, despite there being 11 in the mnemonic. All data were analyzed using SPSS v24.0 (IBM Corp). The mean was used for averages. The Fisher exact test was used when comparing groups of categorical data to provide exact *P* values. *P* values <.05 were considered statistically significant.

## Results

### Baseline Characteristics

Data for 50 consecutive patients with femoral neck fractures were collected during the periods pre-UK lockdown, post-UK lockdown, and after the “MRS BAD BONES” intervention. During data collection, a number of patients were excluded from analysis: 8 patients with periprosthetic fractures, 8 patients who were managed nonoperatively, 12 patients who were taking bone-sparing treatment preadmission, 9 patients for whom it was documented as inappropriate to commence bone-sparing treatment due to a palliative approach, and 14 patients who died while in hospital. The demographics of the patients can be seen in Table 1. The percentage of patients who were reviewed by the orthogeriatric team prelockdown was 88% (n=44), but this fell to 0% following the lockdown, due to redeployment in response to COVID-19 pressures. During the postintervention period, this rose to 38% (n=19) as the orthogeriatric team returned to the T&O wards with the easing of local COVID-19 pressures.

**Table 1:** The demographics of patients with femoral neck fractures whose data were analyzed for this study. The mean ages, the female:male ratio, and the number and percentage of patients who received an orthogeriatric review are shown.

Variable	Prelockdown	Post lockdown	Post intervention
Patients, n	50	50	50
Age (years), mean (SD)	83.8 (8.34)	82.4 (9.16)	82.7 (8.98)
Female:male ratio	33:17	27:23	36:14
Orthogeriatric review, n (%)	44 (88)	0 (0)	19 (38)

The percentage of patients who had blood tests performed per local guidelines, which includes eGFR, adjusted calcium, and vitamin D, was high (96%-100%), with no significant difference between all 3 phases (*P*=.78) (Table 2). However, only 5 PTH blood tests were performed, all in the prelockdown period, with an average of 7.0 (range 4.3-10.8). This represents just 10% of the prelockdown population sample, which is just over 3% of the total sample population.

**Table 2:** Patients receiving blood tests according to guidelines during each phase, which includes vitamin D, parathyroid hormone, adjusted calcium, and estimated glomerular filtration rate (eGFR).

Blood test	Prelockdown	Post lockdown	Post intervention
Vitamin D, n (%)	49 (98)	50 (100)	48 (96)
Adjusted calcium, n (%)	50 (100)	49 (98)	48 (96)
Parathyroid hormone, n (%)	5 (10)	0 (0)	0 (0)
eGFR, n (%)	50 (100)	50 (100)	50 (100)

Average vitamin D, adjusted calcium, and eGFR serum levels for the cohorts can be seen in Table 3. Results show deficiency in serum vitamin D but normal adjusted calcium levels and eGFR, when adjusted for age, sex, and ethnicity.

**Table 3:** Average vitamin D, adjusted calcium, and estimated glomerular filtration rate (eGFR) serum levels.

Blood test	Prelockdown	Post lockdown	Post intervention
Vitamin D (nmol/L), mean (range)	46 (12-113)	37.8 (10-90)	49.1 (10-116)
Adjusted calcium (mg/dL), mean (range)	2.30 (2.06-2.65)	2.28 (2.05-2.54)	2.29 (2.06-2.78)
eGFR (ml/min/1.73 m^2^), mean (range)	80 (18-179)	86 (12-183)	83 (8-156)

### Secondary Prevention of Fragility Fractures

**Figure 3:**
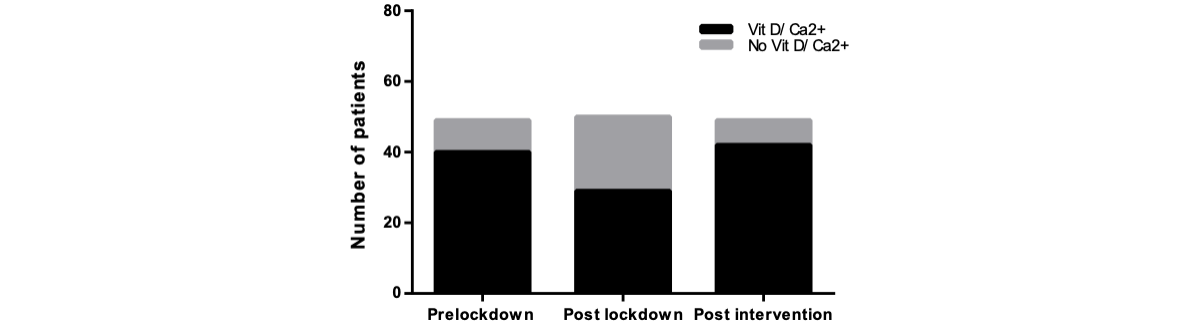
The number of patients receiving correct vitamin D (vit D)/Ca2+ supplements after the lockdown was significantly reduced (*P*<.05); post intervention, there was a significant increase in the correct administration of vitamin D/Ca2+ (*P*<.01), compared to post lockdown.

The purpose of this audit was to assess whether the correct bone health management procedures had been initiated following the UK-wide COVID-19 lockdown. This was broken down into the steps advised by local guidelines, with the following treatments: vitamin D/calcium supplementation, bone-sparing medications, and DEXA scanning. We also noted a subgroup of patients who met the criteria for a DEXA scan but did not have a DEXA requested and instead started bone-sparing treatment. The frequencies and percentages of patients who received the correct osteoporosis management is displayed in Table 4, and Figure 3 to Figure 6.

**Table 4:** Patients who received the correct osteoporosis management procedures during the 3 phases. The patients who met the criteria for a DEXA (dual energy X-ray absorptiometry) scan but were treated with bone-sparing medications instead are also represented.

Osteoporosis management	Prelockdown	Post lockdown	Post intervention
Vitamin D/calcium supplements, n/N (%)	40/49 (81.6)	29/50 (58.0)	42/49 (85.7)
Bone-sparing medication, n/N (%)	17/28 (60.7)	4/22 (18.2)	21/29 (72.4)
DEXA scan, n/N (%)	9/22 (40.1)	1/28 (3.6)	12/21 (60.0)
No DEXA scan; started bone-sparing medication, n/N (%)	9/13 (69.2)	2/27 (7.4)	8/9 (88.9)

**Figure 4:**
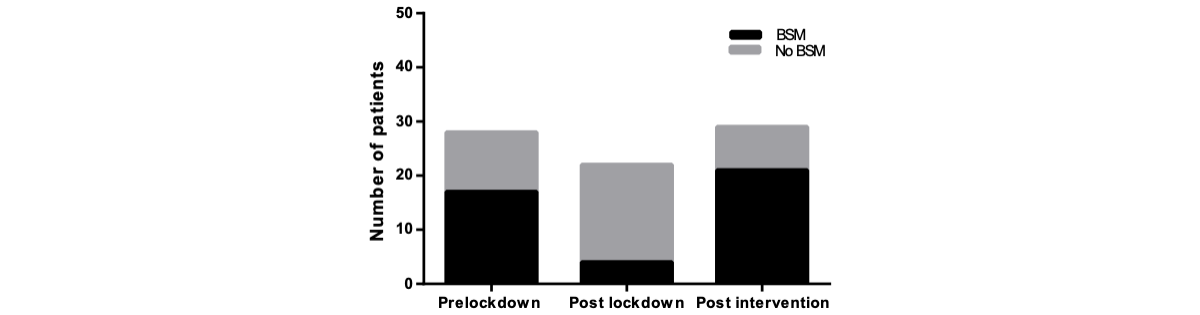
The number of patients receiving correct bone-sparing medication (BSM) after the lockdown was significantly reduced (*P*<.001); post intervention, there was a significant increase in the correct administration of BSM (*P*<.001), compared to post lockdown.

**Figure 5:**
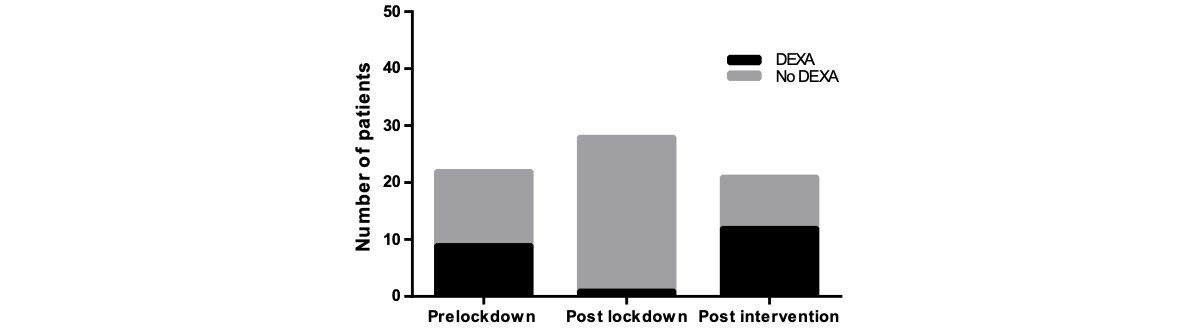
The number of patients being offered a DEXA (dual energy X-ray absorptiometry) scan after the lockdown was significantly reduced (*P*<.001); post intervention, there was a significant increase in patients being requested a DEXA scan (*P*<.001). While numbers of DEXA scans offered increased post intervention compared to the prelockdown period, this was not statistically significant (*P*>.05).

**Figure 6:**
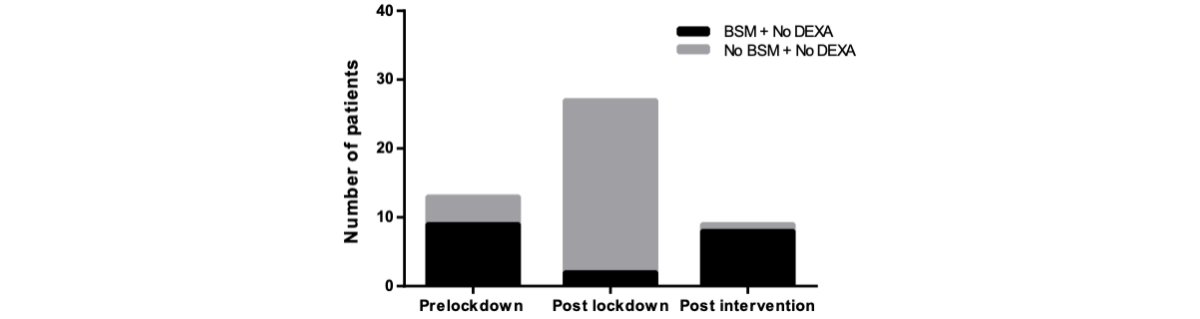
The number of DEXA (dual energy X-ray absorptiometry)-eligible patients who did not have a DEXA scan requested but were started on bone-sparing medication (BSM) significantly dropped following the lockdown (*P*<.001). Post intervention, this significantly increased (*P*<.001), with greater numbers of patients prescribed BSM.

### Vitamin D/Calcium Prescription 

The number of patients receiving the correct vitamin D/calcium treatment following the lockdown was significantly reduced (*P*=.02) with a drop from 81.6% (n=40) to 58% (n=29). Following the implementation of the “MRS BAD BONES” mnemonic, it was found that prescriptions increased to 85.7% (n=42) (*P=*.003), and this was significant when compared to the postlockdown data (Figure 3). However, there was no significant difference between the postintervention and prelockdown findings (*P*=.69).

### Bone-Sparing Medication Prescription

As seen in Figure 4, following the UK lockdown, only 18.2% (n=4) of patients, compared to 60.7% (n=17) of patients prelockdown, were prescribed bone-sparing medication. This represents a significant drop (*P=*.004). However, following the implementation of the “MRS BAD BONES” mnemonic, the prescription of these medications significantly increased to 72.4% (n=21) compared to post lockdown (*P<*.001).

### DEXA Scan Requests

There was a significant drop (*P*=.003) in DEXA scan requests from 40.1% (n=9) to 3.6% (n=1) among eligible patients in the postlockdown period despite a normal service. In the postintervention phase, this number increased to 60% (n=12) and was of significance (*P*<.001) compared to the postlockdown data, as represented in Figure 5. While the percentage of patients with DEXA scans requested increased from 40.1% (n=9) in the prelockdown period, to 60% (n=12) in the postintervention period, this was not statistically significant (*P*=.37)*.* A number of patients were eligible for a DEXA scan as per the guidelines but did not have one requested. Alternatively, they were treated with bone-sparing medication. As seen in Table 3 and Figure 6, prelockdown, 69.2% (n=9) of this subgroup had bone-sparing medication prescribed. Following the lockdown, this dropped to 7.4% (n=2), which was a significant drop (*P*<.001). Post intervention, this significantly increased to 88.9% (n=8) (*P*<.001) when compared to the postlockdown figures.

## Discussion

### Principal Findings

A significant percentage of the patients with a femoral neck fracture who attended UHCW did not receive the correct secondary fragility fracture prevention treatment following the UK lockdown due to the COVID-19 pandemic. Other than PTH, blood testing was performed per guidelines during the 3 phases, but these serum results were not actioned appropriately post lockdown. The redeployment of the orthogeriatric team, who primarily action these results, was the biggest factor leading to this. Clinical management of patients with fragility fractures is often suboptimal even under normal circumstances; a prior study on the secondary prevention of fragility fractures showed that despite steps being taken to increase awareness among junior doctors and nurse practitioners, improvements were still below target [11].

The direct implications on patients who did not receive adequate bone protection are unclear; a follow-up of this cohort may be useful for outcome studies. However, the literature suggests a 10%-13% overall risk of sustaining a secondary contralateral femoral neck fracture, which leads to a 1-year mortality of 31.6% compared to 27.3% for the index fracture. Furthermore, a risk of 28.6% is seen for any other osteoporosis-related fractures. It is clear this injury causes a significant morbidity and mortality among the elderly, as well as financial burden to the NHS [12,13].

The blood tests revealed deficiencies in serum vitamin D, which is to be anticipated in this population [9], but normal adjusted calcium levels and eGFR. No PTH measurements were performed post lockdown, with very few prior; no particular reason for this was identified. Parathyroid pathologies are relatively common endocrine disorders, particularly in elderly women [14]. PTH is vital for the maintenance of calcium homeostasis through its catabolic and anabolic actions that regulate bone remodeling. Hypoparathyroidism can lead to osteoporosis. PTH replacement therapy has been shown to remedy this abnormality [15]. Equally, patients suffering with primary hyperparathyroidism, particularly the normocalcemic variant, have more skeletal complications than is classically seen in hypercalcemic primary hyperparathyroidism. One study found osteoporosis in 57% of a population with normocalcemic primary hyperparathyroidism [16]. Hence, measuring serum PTH, per local guidelines, is important in this cohort and needs to be improved.

NICE recommendations to consider DEXA scanning and treatment of target groups to prevent fragility fractures are based on the extensive evidence that they reduce the risk of hip fractures and are cost-effective when compared to untreated osteoporosis [17,18]. In younger patients, bone mineral density assessment is needed to confirm the diagnosis of osteoporosis since high-energy trauma is often required to fracture the proximal femur. However, appropriate treatment can be commenced in elderly patients with a fragility fracture without the requirement for a DEXA scan [19]. Hence, it is good clinical practice to address this while the patient is still in hospital.

### Strengths

This study highlighted the positive impact on patient care that can be achieved by service changes implemented at a junior level. Following the dissemination of our findings and implementation of the “MRS BAD BONES” mnemonic, there was a significant improvement in the management of bone health compared to the postlockdown period, and was comparable to the prelockdown data. Mnemonics are used in various sectors for teaching purposes. Though used in a hospital environment here, this acronym has the potential to be beneficial in a wider setting, including primary care and medical education. Studies have demonstrated the role of mnemonic strategies in reshaping brain networks and improving memory performance [20]. Finally, though utilized during the COVID-19 pandemic, the use of this mnemonic has the potential to have an ongoing positive impact in the post–COVID-19 era.

### Limitations

Not all parameters included in the “MRS BAD BONES” mnemonic were assessed in this study due to restricted access in requesting archived written documents during the pandemic. Consequently, data were primarily collected using information available from electronic patient records. Those parameters not assessed include medication review, rheumatology/renal advice, smoking cessation, alcohol limits, nutrition, and exercise. The authors opted to use “MRS BAD BONES” despite this limitation because of the importance of each parameter as well as being a memorable acronym that provides a holistic approach to managing fragility fractures as endorsed by NICE. Drugs, particularly glucocorticoids, can induce osteoporosis, which makes a medication review an important assessment. Rheumatology or renal advice can be sought in complex cases where specialist treatment input is required, particularly in patients with severe renal impairment or intolerance of first-line bisphosphonates. Lifestyle changes, including smoking cessation, drinking alcohol within recommended limits, optimizing nutrition, and regular exercise, can all improve bone health and reduce the risk of fragility fractures [17]. Further research may provide insight into whether these factors were significantly affected by the use of the mnemonic. Furthermore, the return of the orthogeriatric team to the orthopedic unit will have contributed to improvements. Due to the unpredictability of the pandemic, their arrival was an unforeseen factor during the intervention phase. However, 62% of the patients in the postintervention phase did not receive an orthogeriatric review because they were discharged prior to their return. Hence, a large proportion of these patients had optimized management prior to the gradual return of services. Another limitation is that the guidelines state all male patients, as well as female patients <75 years old, should have a DEXA scan. Our results showed a significant proportion of patients eligible for DEXA scanning were instead treated with bone-sparing medications. The local guidelines do not take into consideration the experience and clinical judgment made by the orthogeriatric team who on occasions will commence all male patients and female patients <75 years old deemed high risk for osteoporosis onto bone-sparing medications without the need for bone density assessment. An update on the local guidelines to reflect this should be considered when next reviewed. A final limitation of this study is that 50 consecutive patients with femoral neck fractures were collected during 3 phases over a 5-month period. This may be considered a small population over a short duration. A longer period of analysis including the recruitment of patients with nonhip fragility fractures may be useful to further evaluate the findings. Additionally, dissemination of this mnemonic to other orthopedic units might be useful to further validate our results.

### Conclusion

Management of bone health is an important consideration for patients with femoral neck fractures due to the morbidity and mortality osteoporosis poses, and the significant financial burden fragility injuries cause the NHS. Orthogeriatric team input is invaluable in coordinating secondary prevention of fragility fractures. However, with the uncertainty of future COVID-19 outbreaks, subsequent orthogeriatrician redeployment may be required. Despite this, ensuring that fragility fracture management is not forgotten is vital. Here, we present the use of a mnemonic, “MRS BAD BONES,” aimed at junior doctors, advanced nurse practitioners, and medical students, which could be used to improve awareness of major areas of assessment and management of secondary prevention of fragility fractures, and maintain optimal quality of care.

## Abbreviations

DEXA: dual energy X-ray absorptiometry
eGFR: estimated glomerular filtration rate
MRS BAD BONES: medication review, rheumatology/renal advice, smoking cessation; blood tests, alcohol limits, DEXA scan; bone-sparing medications, orthogeriatric review, nutrition, exercise, supplement
NHS: National Health Service
NICE: National Institute for Health and Care Excellence
PTH: parathyroid hormone
T&O: Trauma and Orthopaedics
UHCW: University Hospital of Coventry and Warwickshire
